# Case Report: Pituitary hyperplasia secondary to long-neglected severe primary hypothyroidism: a case of misdiagnosis and lessons learned

**DOI:** 10.3389/fmed.2025.1601190

**Published:** 2025-08-15

**Authors:** Sina Du, Jiawei Sun, Chunnuo Wang, Yanghuan Zhou, Haiying Zhao, Wei Li, Jianwei Chen

**Affiliations:** ^1^Department of Endocrinology, Cixi People Hospital Medical Health Group (Cixi People Hospital), Ningbo, China; ^2^Department of Nephrology, Cixi People Hospital Medical Health Group (Cixi People Hospital), Ningbo, China; ^3^Department of Medical Imaging (Radiology), Cixi People Hospital Medical Health Group (Cixi People Hospital), Ningbo, China; ^4^Department of Clinical Nutrition and Metabolism, Cixi People Hospital Medical Health Group (Cixi People Hospital), Ningbo, China; ^5^Department of Endocrinology, Ningbo Medical Center LiHuiLi Hospital, Zhejiang, China; ^6^Department of Cardiothoracic Surgery, Cixi People Hospital Medical Health Group (Cixi People Hospital), Ningbo, China

**Keywords:** primary hypothyroidism, pituitary hyperplasia, hyperprolactinemia, pituitary macroadenoma, pathology

## Abstract

**Background:**

Primary hypothyroidism is characterized by a loss of thyroxine feedback inhibition and an increase in thyrotropin-releasing hormone (TRH) levels, resulting in reactive pituitary hyperplasia. However, it is important to note that pituitary hyperplasia due to primary hypothyroidism (PHPH) is rare, particularly when symptoms of pituitary mass compression are present.

**Case summary:**

A patient with menstrual irregularities and hyperprolactinemia exhibited pituitary enlargement on magnetic resonance imaging (MRI). Initial treatment with bromocriptine mesylate failed, leading to surgical resection. Preoperative evaluation revealed severe hypothyroidism. Postoperatively, discontinuation of medication resulted in elevated thyroid-stimulating hormone (TSH) levels. Reticulin staining confirmed TSH hyperplasia, likely due to long-standing, untreated hypothyroidism since childhood. Postoperative thyroid hormone therapy restored normal thyroid and pituitary functions.

**Conclusion:**

This case underscores the importance of recognizing long-standing hypothyroidism as a potential cause of pituitary hyperplasia. Accurate diagnosis is essential to avoid unnecessary surgical or pharmacological interventions.

## Introduction

Primary hypothyroidism leads to pituitary hyperplasia (PHPH) is an uncommon endocrine disorder (1). In primary hypothyroidism, low levels of thyroid hormones diminish negative feedback inhibition on the hypothalamus, leading to excessive secretion of thyrotropin-releasing hormone (TRH) and subsequent thyrotropic hyperplasia (2). In addition to the symptoms associated with primary hypothyroidism, PHPH patients may experience local compressive effects due to pituitary hyperplasia, including optic chiasm compression (causing visual field loss and disturbances) and pituitary hormonal disturbances such as hyperprolactinemia (3). Pubertal delay and abnormalities, ovarian hyperstimulation syndrome, pseudoacromegaly, and associated mental and psychological issues may occur (3, 4). PHPH patients respond well to thyroid hormone replacement therapy. In cases of unclear diagnosis, it is essential to avoid making a hasty decision about surgical resection in order to prevent irreversible pituitary dysfunction, which can severely impair the growth and development of adolescents during this crucial period.

Here, we report a case that was misdiagnosed as “TSH pituitary adenoma” across various departments, resulting in the surgical removal of the enlarged pituitary gland. TSH levels were again found to be elevated above the detection limit after the withdrawal of medication. Following thyroid hormone replacement therapy, the patient’s symptoms improved, and the related hormone levels returned to normal. We hope this case of misdiagnosis can serve as a learning opportunity to help others avoid similar mistakes.

## Case presentation

**Figure 1:**
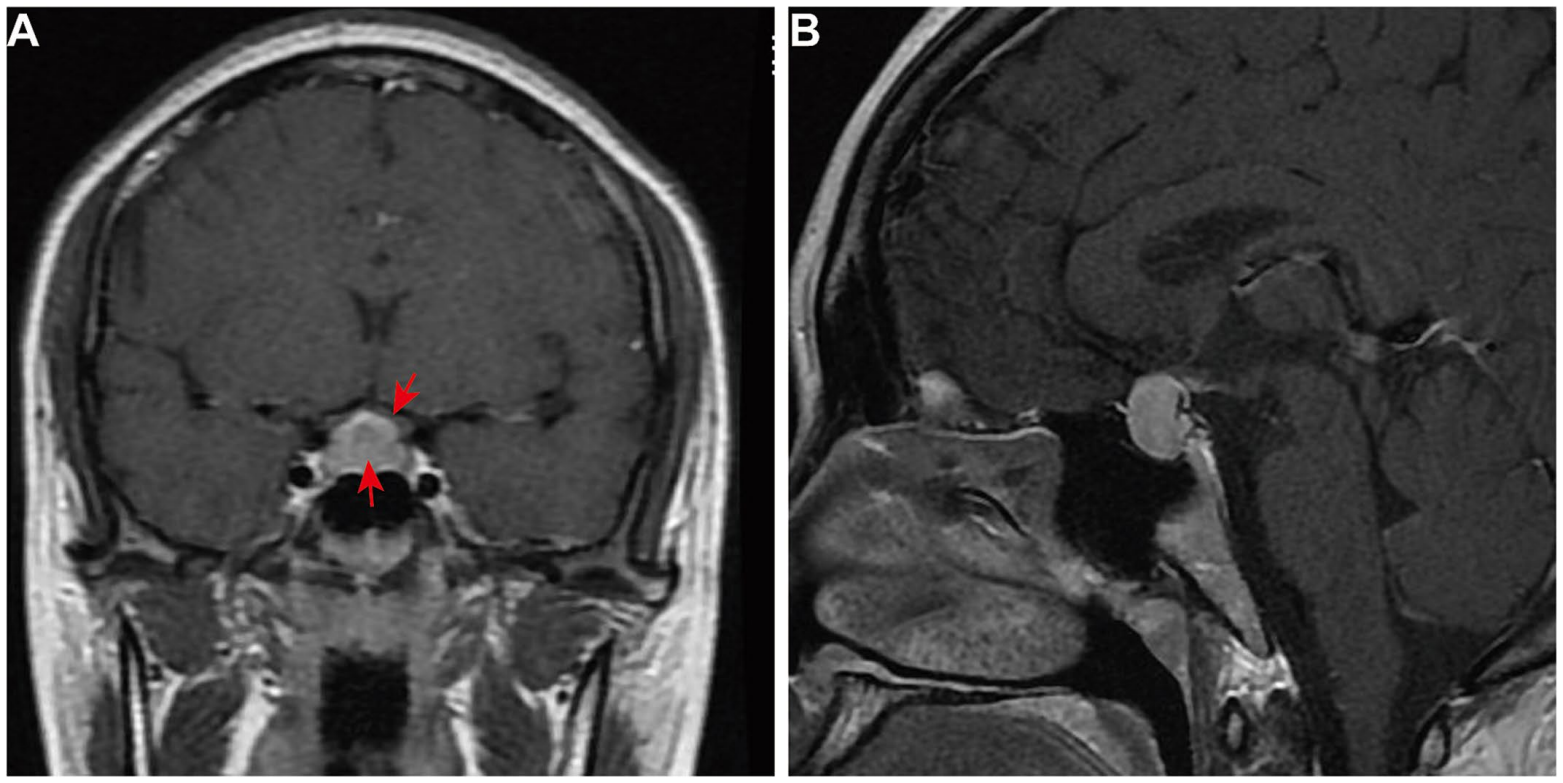
First and preoperative pituitary MRI of the patient. The contrast-enhanced T1-weighted MRI images of the pituitary gland include **(A)** coronal view and **(B)** a sagittal view. These images show an enlarged and homogeneously enhanced pituitary gland (arrow), measuring 1.9 cm × 1.6 cm × 0.8 cm. There is a mass effect on the optic chiasm (arrow).

On 22 June 2017, a 22-year-old female patient presented to the gynecology department with headache, dizziness, blurred vision, and prolonged menstrual cycles. The prolactin (PRL) level was 2,554 mIU/L (ref: 59–619 mIU/L). Magnetic resonance imaging (MRI) of the pituitary gland revealed an enlarged pituitary fossa containing a mass (8 mm × 19 mm × 16 mm). This mass demonstrated homogeneous signal intensity on both T1-and T2-weighted sequences, with significantly uniform enhancement, suggestive of pituitary macroadenoma (Figures 1A, B). She was treated with bromocriptine mesylate (1 mg twice daily) for 3 months. However, her symptoms showed no significant improvement, her PRL level remained elevated, and follow-up MRI revealed no notable reduction in the size of the pituitary lesion.

**Figure 2:**
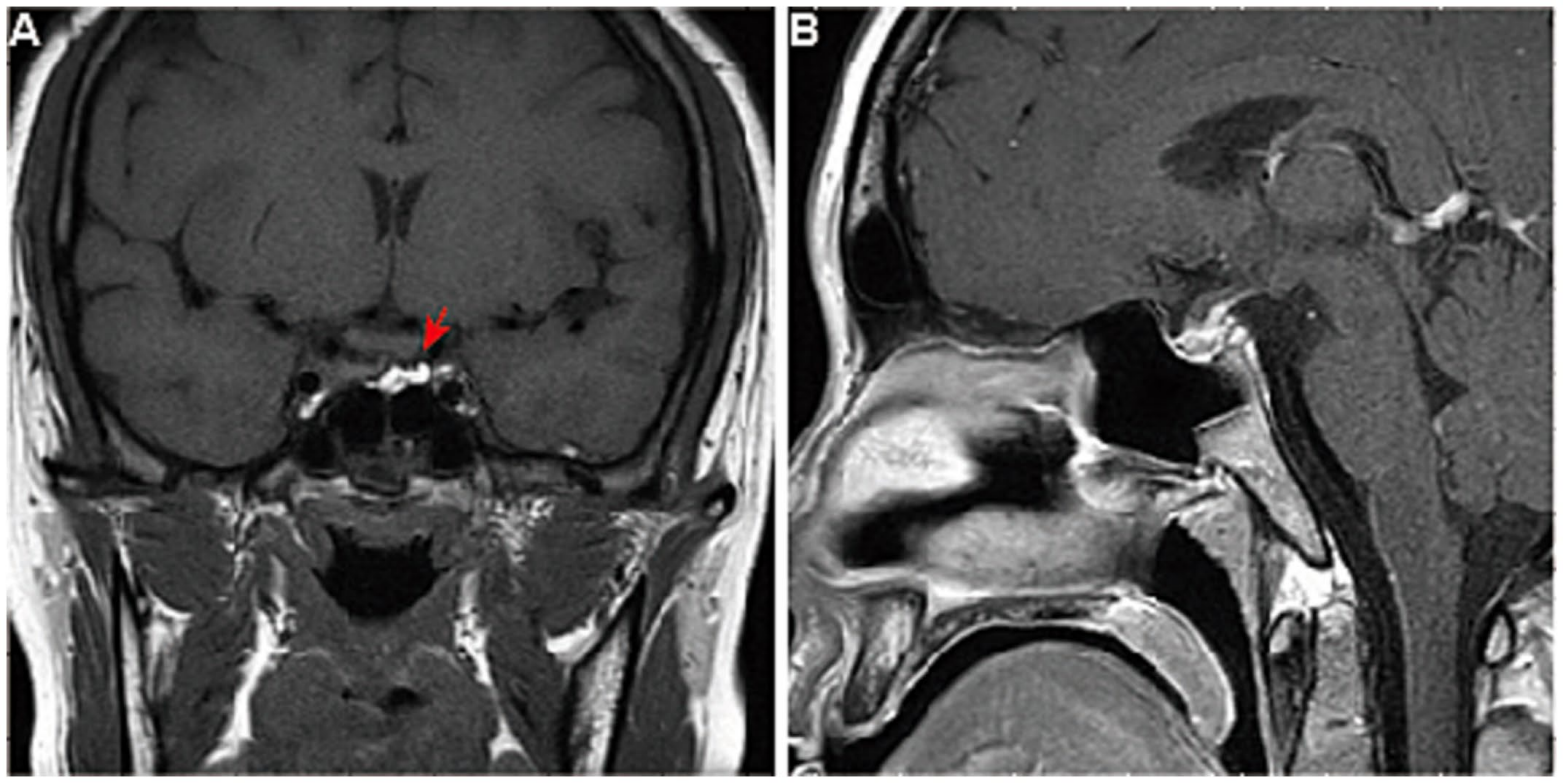
Pituitary MRI of the patient after surgery. The pituitary gland of the patient after surgery, viewed in **(A)** coronal and **(B)** sagittal planes of enhanced T1-weighted MRI, showed partial resection of the sellar mass without an optic chiasma compression (arrow).

**Figure 3:**
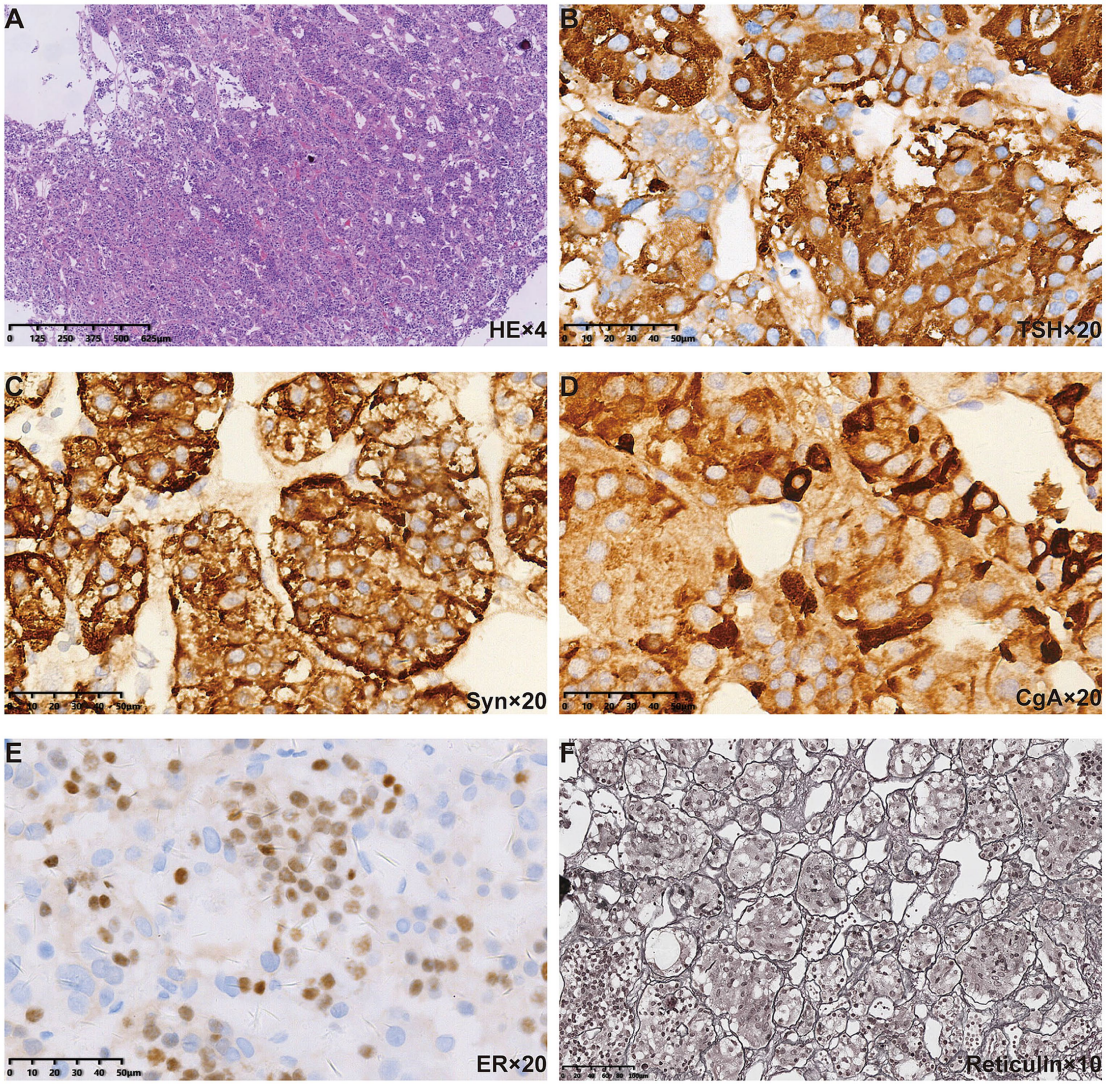
Pituitary pathological findings of the patient. **(A)** HE staining revealed polymorphous cell population arranged in acinar architecture. Positive immunostaining for **(B)** TSH, **(C)** Syn, and **(D)** CgA was found in the cytoplasm. **(E)** Positive immunostaining for ER was found in the nuclei. **(F)** Reticulin staining further revealed the normal acinar architecture.

The patient then presented to the department of neurosurgery for surgical resection and underwent an evaluation before the planned surgery. On 26 October 2017, thyroid function tests suggested elevated levels of thyroid-stimulating hormone (TSH), and decreased levels of tetraiodothyronine (T4), and free T4 (Table 1). Serum sex hormones tests revealed elevated PRL levels, while plasma adrenocorticotropic hormone (ACTH) and cortisol (COR) levels were normal (Table 2). Blood routine and urine tests, as well as assessments of coagulation function, electrolyte, liver, and kidney function, were normal. Thyroid Doppler ultrasonography suggested the presence of Hashimoto thyroiditis (HT). She underwent surgery after receiving thyroid hormone replacement therapy. Post-operative pituitary MRI showed partial resection of the sellar mass (Figures 2A, B), and the levels of TSH and PRL decreased significantly (Tables 1, 2). The pathological findings from the initial draft were diagnostic of “TSH pituitary adenoma.” Immunohistochemistry results were positive for ER (estrogen receptor), Ki-67 (index approximately 1%), TSH, Syn (synaptophysin), CgA (chromogranin A), and were negative for P53, ACTH, PRL, GH (growth hormone), LH, FSH (Figures 3A–E).

**Table 1:** Thyroid function test results of the patient.

Date	T3 (ref: 1.02–2.96 nmol/L)	T4 (ref: 55.47–161.25 nmol/L)	FT3 (ref: 2.77–6.31 pmol/L)	FT4 (ref: 10.45–24.38 pmol/L)	TSH (ref: 0.550–4.78 mIU/L)
2017-10-26	1.14	30.4	2.9	5.11	>150
2017-10-31	0.71	43.7	2.2	6.27	39.16
2017-11-10	0.78	96.2	2.6	14.69	33.30
2017-11-16	0.5	88.6	3	14.02	18.19
2017-12-05	1.56	92	4.21	15.28	18.60
2018-01-28	0.57	18.2	2.25	4.33	>150
2018-03-07	1.87	109	5.12	16.5	2.35
2018-04-19	1.62	111.9	4.77	14.29	2.96
2018-06-30	1.31	110.2	4.08	15.24	8.73
2018-09-07	1.56	117	5.08	16.7	0.62
2019-04-17	1.25	83.8	3.75	11.98	48.13
2020-05-04	1.54	145	4.92	16.88	1.86
2020-08-24	1.5	156.4	4.9	20.52	0.97
2024-08-09	/	/	3.14	9.91	123.59
2024-12-01	0.89	56.43	1.91	6.48	58.76

/: not available; T3: triiodothyronine; T4: tetraiodothyronine; FT3: free triiodothyronine; FT4: free tetraiodothyronine; TSH: thyroid-stimulating hormone.

**Table 2:** Laboratory data of the patient.

Date	FSH (ref: 2.5–10.2 IU/L)	LH (ref: 1.9–12.5 IU/L)	PRL (ref: 59–619 mIU/L)	E2 (ref: 71.6–529.2 pmol/L)	T (ref: 0.5–2.6 nmol/L)	P (ref: <4.45 nmol/L)	ACTH 8:00 (ref: 0–46 pg./mL)	ACTH 16:00 (pg/ml)	COR 8:00 (ref: 43–224 ng/mL)	COR 16:00 (ref: 30.9–166.6 ng/mL)	Na (ref:135–145 mmol/L)	GH (ref: 0.126–9.88 ng/mL)
2017-10-26	2.97	3.59	1011.8	301	1.41	25.9	36.4	6.2	170.8	43.73	/	/
2017-10-31	1.03	1.9	97.9	497	1.29	49.58	10.6	5.43	34.55	78.97	122.1	/
2017-11-10	/	/	116.5	/	/	/	/	/	/	/	135.8	/
2017-11-16	4.28	4.36	132.4	196	0.64	0.68	8.14	8.01	97.4	42.59	/	/
2017-12-05	/	/	104.9	/	/	/	23.9	20.7	112.48	31.36	/	/
2018-03-07	4.25	5.01	106.7	194	1.26	13.2	21.8	21.3	51.15	74.3	142.3	/
2018-04-19	/	/	/	/	/	/	48.3	34.1	198.56	89.74	137	0.058
2018-06-30	3.75	2.52	86.8	154	1	3.95	/	/	/	/	/	/
2018-09-07	5.8	6	75.3	226	1.67	1.49	25	25.7	88.87	99.35	/	/
2019-04-17	4.25	5.12	140.8	243	2.05	9.29	42.9	27.6	154.05	64.12	/	/
2020-05-04	1.99	4.13	94.7	483	0.98	94.95	37.7	16.1	162.58	44.85	/	/
2020-08-24	0.61	2.41	126.4	471	0.62	31.42	/	/	/	/	137.3	/
2024-08-09	2.86	1.39	265	280.6	0.72	4.77	54.04	22.68	178.17	69.89	139.7	/

/: not available; FSH: follicle-stimulating hormone; LH: luteinizing hormone; PRL: prolactin; E2: estradiol; T: testosterone; P: progesterone; ACTH: adrenocorticotropic hormone; COR: cortisol; GH: Growth Hormone.

After discharge, the patient mistakenly believed that the “pituitary macroadenoma” had been resected and therefore deemed medication unnecessary, discontinuing levothyroxine for a full month. On 28 January 2018, routine follow-up revealed that the TSH level was again above the detection limit (Table 1). At this point, we noted that the patient had been diagnosed with a thyroid-related disorder at the age of 10 but had received neither consistent treatment nor regular follow-up monitoring. Physical examination showed facial edema, blurred vision, and slow reflexes. Despite having two uneventful pregnancies, she had not undergone systematic prenatal care. Given the presence of severe hypothyroidism both before and after surgery, we questioned the correctness of the previous diagnosis of “TSH pituitary adenoma.” Upon re-evaluation of the patient’s preoperative pituitary MRI, we observed diffuse and homogeneous enlargement of the pituitary gland, with suprasellar extension that exhibited a dome-shaped contour. Reticulin staining showed no evidence of loss of normal acinar architecture to confirm a diagnosis of PitNET (Figure 3F). Consequently, the diagnosis was revised to PHPH.

**Figure 4:**
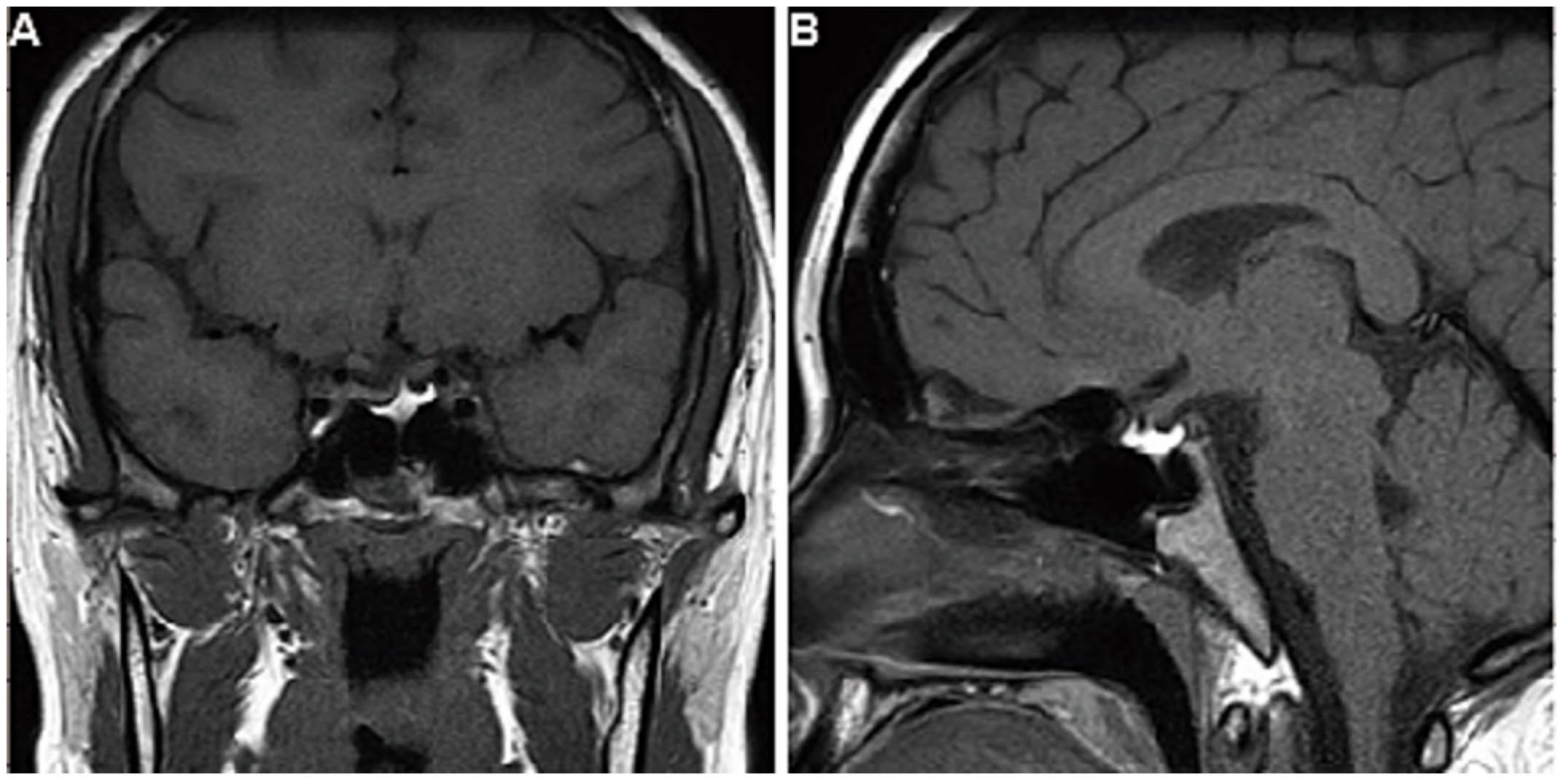
Follow-up imaging findings of the patient. After 7 years of follow-up, the contrast-enhanced T1-weighted MRI images of the pituitary gland, viewed in **(A)** coronal and **(B)** sagittal planes, showed no expansion of the residual gland.

The patient was prescribed levothyroxine (100 mg daily) and followed for 7 years. Her thyroid hormone levels remained stable with medication, while they fluctuated without it (Table 1). Serum levels of ACTH, COR, GH, sex hormones, and sodium were consistently within the normal range (Table 2). The pituitary MRI showed no expansion of the residual gland (Figures 4A, B).

## Discussion

In primary hypothyroidism, low levels of thyroid hormone reduce hypothalamic negative feedback, leading to TRH hypersecretion (2). Elevated TRH levels drive thyrotroph hyperplasia while concurrently stimulating lactotroph expansion, which results in hyperprolactinemia (4). HT is considered to be the most common cause of primary hypothyroidism (1). Research indicates that patients with TSH levels above 100 μIU/mL have nearly 10 times the risk of pituitary enlargement compared to those with TSH levels between 50 and 99 μIU/mL (5–8). The prevalence of hyperprolactinemia in primary hypothyroidism varies from 0 to 42%, with the degree of hyperprolactinemia correlated to the severity of hypothyroidism (4).

PHPH is occasionally misdiagnosed as a pituitary macroadenoma (9). On MRI, PHPH typically shows homogeneous, symmetric anterior pituitary enlargement with a dome-shaped contour, enhanced T1/T2 signals, and the absence of hemorrhage, necrosis, or cystic changes (10, 11). The lesion may progress rapidly after the onset of hypothyroidism (12), with possible suprasellar extension (gourd-like appearance) and optic chiasm compression; however, it rarely invades the cavernous sinus (10). In contrast, pituitary adenomas exhibit mixed signal intensities (13), often distort the pituitary stalk, and frequently invade surrounding structures such as the sphenoid sinus, cavernous sinus, and internal carotid artery (10).

Hyperplasia features lobular cell arrangements with dominant immunopositivity for one hormone. Reticulin staining is the most useful method for distinguishing adenomas from normal adenohypophysis parenchyma and hyperplastic lesions. The normal pituitary gland retains a reticulin pattern, which is increased in cases of pituitary hyperplasia and disrupted in pituitary adenomas (14, 15). The diagnosis of PHPH in our case was confirmed through reticulin staining.

Thyroid hormone replacement therapy is the primary method for treating PHPH. During the entire treatment process, serum levels of T3, T4, and TSH should be routinely evaluated. Pituitary size reduction should be observed after levothyroxine treatment. Studies have shown that when PHPH patients are followed up, 71.4% patients have a full resolution of pituitary hyperplasia. In 22.1% of cases, there has been at least a documented reduction in pituitary size (4). If TSH levels partially decrease without significant improvement in thyroid function after 3 months of replacement therapy, the possibility of long-term increased secretion of TSH-secreting cells in the anterior pituitary should be considered, which may lead to adenoma formation. If, after 6 months of thyroxine therapy, TSH levels decline but PRL levels remain elevated, accompanied by persistent amenorrhea, galactorrhea, and no reduction in pituitary size, a pituitary PRL adenoma should be suspected (16).

Adolescent children with PHPH may experience local compression symptoms produced by pituitary hyperplasia and the related levels of pituitary hormones (3). Pubertal delay and abnormalities, as well as associated mental and psychological issues, may occur (3). The patient had pituitary hyperplasia secondary to long-term severe hypothyroidism. Based on the medical history, we suspected this condition may be related to hypothyroidism that developed before puberty and remained unmonitored and untreated.

Preoperative adrenal and gonadal functions were normal. Menstrual irregularities can be improved with hypothyroidism control, requiring no intervention. However, the patient’s symptoms of headache, dizziness, blurred vision, and prolonged menstrual cycles aggravated. Pituitary MRI showed no significant reduction in pituitary volume following bromocriptine treatment. She underwent surgery due to a poor response to bromocriptine. Intraoperatively, the surgeon found the lesion had a massive adhesion and a tough texture. The lesion had compressed the surrounding normal tissues, making it difficult for the patient to avoid surgery. In the course of follow-up, the patient became pregnant and underwent a two-time induced abortion. Blood pressure, reproductive hormone levels, thyroid function, and adrenal function were all normal, indicating no significant impact on sexual or reproductive health. Unfortunately, we did not pay enough attention to the severe hypothyroidism detected before surgery, and she was rushed into surgery without standardized thyroid hormone replacement therapy. PHPH patients should undergo receive hormone replacement therapy. If symptoms improve with normalization of hormone levels and pituitary size reduction, surgical intervention can be avoided. Thyroid levels stabilized with levothyroxine but fluctuated when she self-discontinued the medication. Primary hypothyroidism requires treatment with levothyroxine; otherwise, high levels of TSH may be observed along with the risk of pituitary hyperplasia. Improved communication and standardized management are crucial to enhance patient understanding, compliance, and treatment outcomes.

## Conclusion

Severe hypothyroidism rarely causes pituitary hyperplasia with mass effect and clinical symptoms. All cases of hyperprolactinemia should be evaluated for thyroid hormone levels. This highlights the importance of screening for thyroid function in patients with pituitary imaging abnormalities, as pituitary hyperplasia can be effectively reversed with thyroid hormone replacement therapy. However, if there is no response to levothyroxine therapy, it is crucial to consider the possibility of pituitary adenomas. This approach ensures appropriate management and helps prevent misdiagnosis.

## Data Availability

The original contributions presented in the study are included in the article/supplementary material, further inquiries can be directed to the corresponding author/s.
